# Epidemiology, Bacteriological Profile, and Antibiotic Sensitivity Pattern of Burn Wounds in the Burn Unit of a Tertiary Care Hospital

**DOI:** 10.7759/cureus.4794

**Published:** 2019-06-01

**Authors:** Noman A Chaudhary, Muhammad D Munawar, Muhammad T Khan, Kausar Rehan, Abdullah Sadiq, Ahsan Tameez-ud-din, Hamza Waqar Bhatti, Zuhair Ali Rizvi

**Affiliations:** 1 Surgery, Rawalpindi Medical University, Rawalpindi, PAK; 2 Internal Medicine, Rawalpindi Medical University, Rawalpindi, PAK; 3 Pathology and Microbiology, Benazir Bhutto Hospital, Rawalpindi, PAK; 4 Miscellaneous, Rawalpindi Medical University, Rawalpindi, PAK

**Keywords:** epidemiology, burns, wounds, bacteria, antibiotic

## Abstract

Introduction: Burn wounds are commonly infected by organisms which delay wound healing. Therefore, it is necessary to evaluate the flora obtained from wounds of burn patients in order to determine the most effective treatment. The aim of this study was to determine the frequencies of various bacteria isolated from burn wounds and to determine their antimicrobial susceptibility.

Materials and methods: This descriptive cross-sectional study was conducted from January 2018 to November 2018 which included consecutive samples of burn wounds from patients admitted to the burn ward of a tertiary care hospital. Bacteria and their antimicrobial susceptibility were determined by swab cultures and sensitivity tests by standard aseptic techniques. Data were analysed via the Statistical Package for Social Sciences (SPSS), v23.0 (IBM SPSS Statistics, Armonk, NY). Chi-square tests were applied between qualitative variables, while the Kruskal-Wallis test was applied to compare the means of asymmetrical data. Bacterial isolates and their susceptibility pattern were represented as frequencies and pie charts.

Results: A total of 178 samples were obtained from 109 patients from burn wounds. One hundred and twenty-two wounds (68.5%) showed growth and 56 (31.4%) showed no growth after 24 hours of incubation. Positive cultures were significantly more frequent in wounds of greater than one-week duration (p < 0.002). Out of 158 bacterial isolates, the most common isolate was *Pseudomonas aeruginosa* - 41 specimens (24.91%), followed by *Staphylococcus aureus* - 38 specimens (24.05%), *Acinetobacter* - 27 (17.09%), *Klebsiella* - 24 (15.19%), *Escherichia coli* - 13 (8.23%), *Proteus* - 7 (4.43%), other coliforms - 6 (3.8%), *Enterococcus* - 1 (0.63%), and Enterobacter - 1 (0.63%). Drug resistance to penicillin G, ampicillin, Augmentin, ceftazidime, cefotaxime, ceftriaxone, meropenem, and piperacillin+tazobactam was exceptionally high.

Conclusion: The most common bacterial isolates are *Pseudomonas aeruginosa* and *Staphylococcus aureus*. Piperacillin+tazobactam against *Pseudomonas aeruginosa* and vancomycin and linezolid against *Staphylococcus aureus* are highly effective and can be used as empirical therapies.

## Introduction

Burn injuries to the skin and other tissues are caused by heat, radiation, electricity, friction, or contact with chemicals [1]. Around 180,000 deaths occur annually due to burns, and the majority of these deaths occur in low or middle income developing countries [1]. They are the leading cause of disability-adjusted life years (DALYs) lost in these countries. Children and females are the two major groups who become victims of burn injuries. In Bangladesh, Colombia, Egypt, and Pakistan, 17% of children with burns have a temporary disability and 18% have a permanent disability. Burn injuries which do not cause death result in a significant amount of morbidity, prolonged hospitalization, disfigurement, and disability, often with resulting stigma and rejection [1]. Globally in 2015, fire and heat resulted in 67 million injuries [2].

Burn wounds are a susceptible site for the growth of opportunistic organisms of endogenous and exogenous origin [3]. Infection in the burn patient is an important cause of morbidity and mortality and presents a challenge for the burn team [4]. There is an increased risk of infections due to altered immunity which may lead to sepsis [5]. Complications due to infections account for 50% - 75% of mortality in burn patients after initial care [6-8]. There are many factors which lead to infections in burn patients, such as exposed body surface, immunocompromised state, invasive procedures carried out in the health care facility, and prolonged hospital stay. Factors related to a patient, such as age, total body surface area (TBSA), and depth of burn wound, and factors related to microbiological organisms, such as type and number, enzyme/toxin production, and motility of organisms, both determine invasive infection. Superficial bacterial contamination can cause sepsis in burn patients and both are directly correlated with one another [8-10]. The burn wound has three characteristic areas. The first one is the zone of coagulation nearest to the source of heat which forms an eschar. Adjacent to this area is the zone of stasis which is at increased risk for ischemia due to loss of perfusion. The outermost zone, the zone of hyperaemia, consists of relatively normal skin with increased blood flow and vasodilation and minimal area of cellular injury. The most important is the area containing moist protein-rich eschar, which supports microbial growth due to its avascular nature and hinders the delivery of immune cells and antibiotics [6]. The burn wound remains sterile immediately after thermal injury [3], but after that, it becomes rich in organisms transferred by the hands of health care personnel and fomites [11]. The gastrointestinal tract is an important source of organisms in burn patients and these endogenous organisms may be transmitted to the surface by faecal contamination of wounds [11].

An increase in the patient load in government hospitals causes a delay of a few days between ordering the cultures and receiving culture reports. During this crucial time, empirical antibiotic therapy can be given to the patients to control the infection. However, due to the inappropriate prescription of antibiotics [12] and their overuse [12-13], antibiotic resistance is increasing [14]. Therefore, this study was conducted to find the most common pathogens and the appropriate antibiotic for the microbes isolated from the wounds of the burn patients to be used as empirical and definitive therapy.

## Materials and methods

This descriptive cross-sectional study was conducted in the burn unit of a tertiary care hospital. The Institutional Research Forum, Rawalpindi Medical University issued approval RSRS-2018-P-01. The study was conducted from January 2018 to November 2018 using consecutive non-random sampling. We included male and female patients of all ages who were admitted to the burn unit of our hospital. Patients who were admitted again after being discharged once, either for follow-up or for plastic surgery procedures, were excluded. One hundred and seventy-eight culture reports of 109 patients were included. Patients who had a prolonged hospital stay had multiple culture reports, while those who had minor burn injuries and short hospital stays had only one culture report at the time of hospital admission. All patients had their wound cultures and sensitivities done at the time of admission. Samples were collected from burn wounds by culture swabs under aseptic techniques. They were then sent to microbiology laboratory where samples were inoculated on agar plates. Wound culture was done on blood and McConkey agar. Cultures were incubated at 37°C for 24 - 48 hours. Microbes were identified under the microscope by observing morphological characteristics and applying biochemical tests. Antibiotic sensitivity pattern was done on Muller Hinton agar by Kirby Bauer disk diffusion method. Age, gender, total body surface area burnt (TBSA), type of burn, and date of admission were collected from the patients' record.

Data was entered and analysed using the Statistical Package for Social Sciences (SPSS), v23.0 (IBM SPSS Statistics, Armonk, NY). Descriptive statistics were applied to find frequencies, percentages, means, and standard deviations. Quantitative variables, such as age and TBSA, were expressed as means and standard deviations. Qualitative variables, such as type of bacteria and type of burn injuries, were expressed as frequencies and percentages. Normality of quantitative variables was checked by applying Kolmogorov-Smirnov and Shapiro-Wilk tests. Independent samples Mann-Whitney U test and Kruskal Wallis test were applied to find a significant difference between means of non-parametric variables having two and more than two categories, respectively. Chi-square tests were applied to compare the percentages of two categorical variables. Level of significance was set at p < 0.05.

## Results

A total of 178 culture reports from 109 patients. Sixty patients (55%) were males, while 49 patients (45%) were females. Ages ranged from two years to 75 years. The median age was 21 years (interquartile range (IQR) = 25.75). The percentage of burns ranged from 3% to 95%. The median TBSA was 30% (IQR = 23.50). Most of the burns encountered were due flame - 70 (64.2%), followed by electric burns - 20 (18.3%), scald - 16 (14.7%), acids - 1 (0.9%), and alkalis - 1 (0.9%). The type of burn was missing for one patient (0.9%). Flame burns were significantly more frequent in females (52.9%) than in males (47.1%), while scalding and electric burns were significantly frequent in males (56.3% and 85%) than in females (43.8% and 15%, respectively) (p < 0.03). The results of independent samples Kruskal-Wallis test showed that the distribution of age was significantly different among the type of burn injury. Scald burns were significantly more frequent in children, while flame and electric burns were more frequent in adults (p < 0.000). 

Out of 178 culture reports, 122 (68.5%) showed positive growth while the remaining 56 (31.5%) showed negative growth. Positive growth was significantly more frequent after the first week of hospital stay (p < 0.002). Positive growth was also more frequent in burn injuries due to causes other than flame (78.9%) than in flame injuries (61%) (p = 0.012). Out of 122 positive growths, 88 (49.4%) had a single growth while 34 (19.1%) showed polymicrobial growth. 

**Figure 1:**
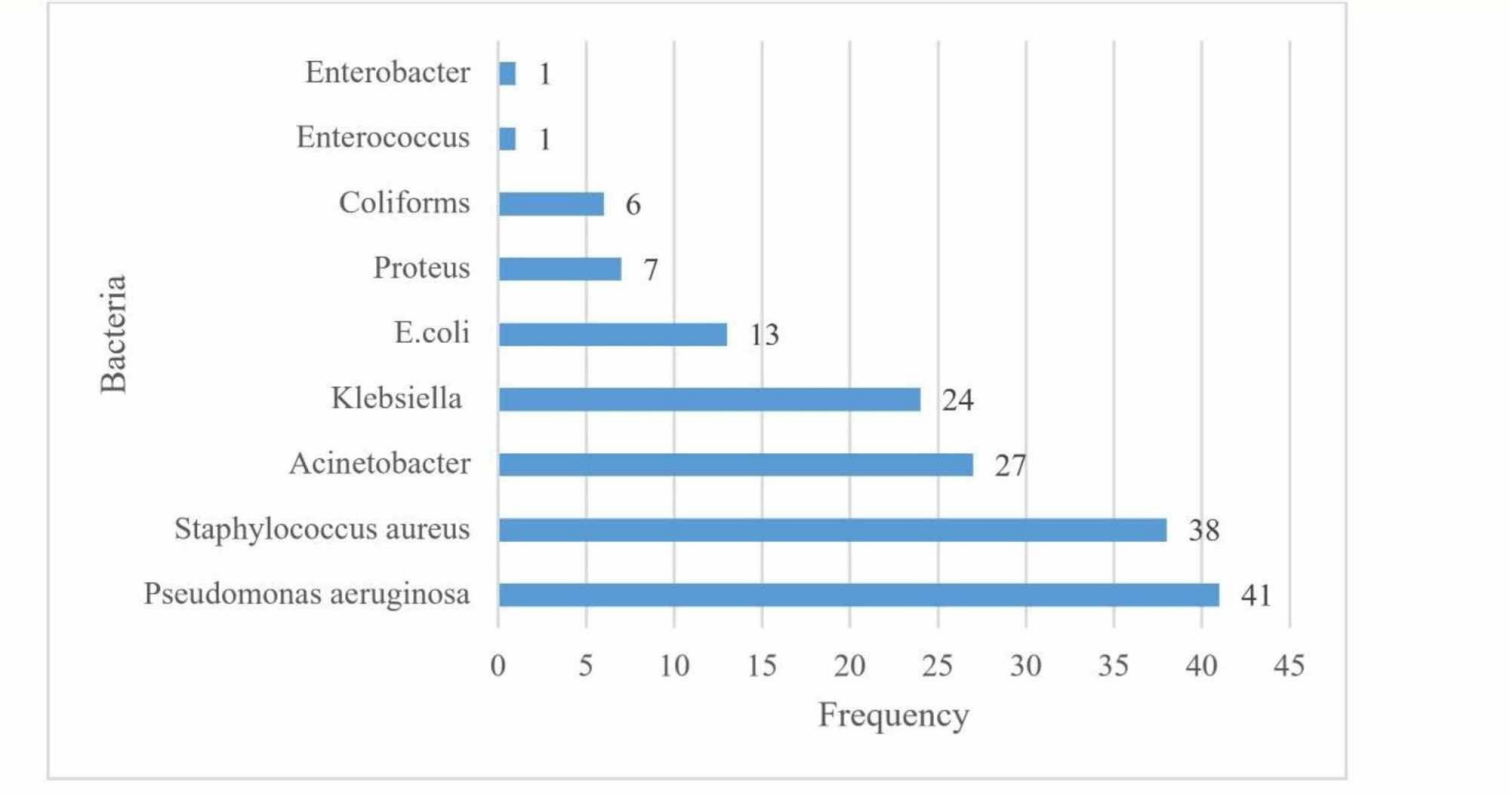
Number of bacterial isolates n = 158

A total of 158 bacterial isolates were recorded from 122 positive growths (Figure 1).

**Figure 2:**
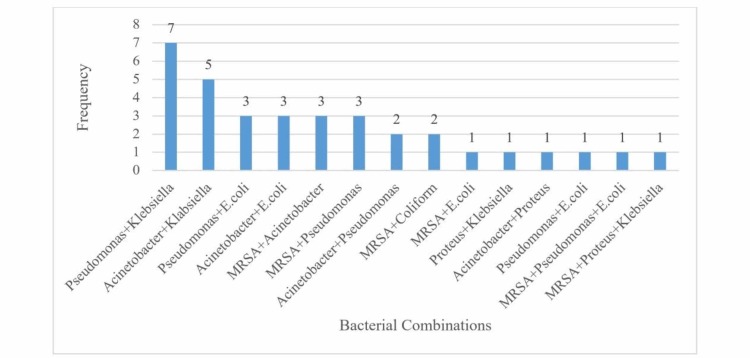
Pattern of mixed growth n = 34 MRSA: methicillin-resistant Staphylococcus aureus

Out of these, 119 (75.31%) bacterial isolates were gram-negative bacilli, while 39 (24.68%) were gram-positive cocci. The most frequent bacterial isolate was *Pseudomonas aeruginosa (P. aeruginosa)* - 41 (24.95%), followed by *Staphylococcus aureus (S. aureus)* - 38 (24.05%). In third place was *Acinetobacter* - 27 (17.09%), followed by *Klebsiella* - 24 (15.19%), *Escherichia coli (E. coli)* - 13 (8.23%), *Proteus* - 7 (4.43%), other - 6 (3.8%), *Enterococcus* - 1 (0.63%), and *Enterobacter* - 1 (0.63%). Twenty-seven out of 38 (71.05%) *S. aureus* isolates were methicillin-resistant *S. aureus* (MRSA). Five out of 41 (12.2%) of the *P. aeruginosa* were pan-resistant. The pattern of growth is shown in Figure 2.

The antimicrobial sensitivity pattern of bacterial isolates is shown in Table 1.

**Table 1:** Antibiotic sensitivity pattern of bacterial isolates from burn wounds

Antibiotics tested	Pseudomonas aeruginosa (n = 41)	Staphylococcus aureus (n = 38)	Acinetobacter (n = 27)	Klebsiella (n = 24)	Escherichia coli (n =13)	Proteus (n = 7)	Coliforms (n = 6)
Piperacillin + tazobactam	38.9%	16.7%	0%	20.8%	54.5%	80%	50%
Amikacin	25%	60%	0%	20.8%	38.5%	33.3%	16.7%
Imipenem	23.3%	10%	-	44.4%	66.7%	80%	50%
Meropenem	19%	14.3%	-	40%	50%	50%	25%
Aztreonam	19.4%	-	-	-	-	-	-
Vancomycin	-	100%	-	-	-	-	-
Linezolid	-	100%	-	-	-	-	-
Cefotaxime	11.5%	10.7%	4.3%	5%	0%	0%	0%
Ceftazidime	8.8%	10.3%	4.3%	5.6%	0%	-	0%
Ceftriaxone	6.9%	14.8%	10%	5%	0%	0%	0%
Cefepime	8.3%		-	-	16.7%	-	-
Methicillin	-	15.4%	-	-	-	-	-
Amoxicillin clavulanate (Augmentin)	-	16.1%	5.3%	0%	0%	0%	-
Ciprofloxacin	-	9.5%	0%	15%	55.6%	25%	33.3%
Moxifloxacin	7.1%	58.3%	0%	-	-	-	-
Cefoperazone + Sulbactam (Sulzone)	-	-	17.6%	23.5%	50%	40%	-
Tigecycline	-	-	96%	-	-	-	-

## Discussion

The skin is a protective physical barrier against microorganisms which are disrupted in burn injuries. The disruption of the vasculature below the skin also plays an important role in the growth of microorganisms in burn wounds [6]. According to the American Burn Association, there is a spectrum of infections in burn wounds consisting of wound colonization, wound infection, invasive infection, and infection/fasciitis [15]. Wound colonization is defined as the presence of a low concentration of bacteria on the surface without invasion or systemic manifestations. When there is more than 10^5^ of tissue in the wound, we call it a wound infection. When more than 10^5^ of tissue in the burn wound causes the formation of pus and separation of the eschar, loss of graft with the involvement of tissue, or the presence of systemic sepsis, then it is called invasive infection. Cellulitis is erythema, induration, warmth, and tenderness in the tissue surrounding the burn wound, and when invasive infection involves the deeper structures below the skin, then it becomes a necrotizing infection [8, 15].

In our study, more males (55%) were affected by burn injuries as compared to females (45%). This is in accordance with the study conducted at the Burn Care Centre of the Pakistan Institute of Medical Sciences (PIMS), Islamabad by Saaiq et al. where 53.68% were males [16]. However, according to another study conducted in India by Datta et al., more females (76.4%) were affected as compared to males (23.6%) [17]. The reason for this can be that males are exposed to occupational hazards and are thus at a greater risk of encountering burn injuries than females. The most common cause of burn injuries differed in males and females. Flame burns were significantly more frequent in females as most of the flame injuries are acquired domestically and most of the women in our society are involved in daily household work in the kitchens where there is an increased risk of flame burns, whereas electric and scald burns were more frequent in males due to occupational hazards. The cause of burn injuries also showed a significant pattern in different age groups. Scald burns were mostly encountered in children because they frequently come in contact with hot liquids like milk and water, while electric burns were more frequent in adults because adults are more conscious about their children coming in contact with fire and electric equipment. Our study consisted of patients of all age groups (from two years to 75 years), while patients in the study by Saaiq et al. ranged in age from one year to 55 years [16]. In our study, admitted patients had TBSA from 3% to 95%, whereas TBSA in the study by Saaiq et al. was from 5% to 40%. Flame burns were the most common cause of burn injuries in our study which is similar to the study conducted by Saaiq et al. After flame injuries, we encountered electric and scald injuries as common causes of burns. This was in contrast with the study by Saaiq et al. in which electric and scald burns were the second and third most common cause of burns. The increased number of flame burns were due to the increased number of gas leakage incidents and explosions in winter.

We found that 68.5% of the cultures showed positive growth. This was in contrast with the higher rate of infection in the studies conducted in India where 88.2% and 93% of the samples showed positive growth [17-18]. Positive cultures were significantly frequent after the first week of stay in the hospital. On the contrary, in the study conducted by Saaiq et al., positive cultures were significantly more frequent after two weeks of injury [16]. The increased number of positive cultures after the first week of hospital stay shows a higher rate of nosocomial infections in our setting. Positive growth was significantly more frequent in flame injuries (78.9%) than that in injuries due to other causes (61%). These results are statistically significant but are not clinically so important.

In our study, *P. aeruginosa* was the most common pathogen isolated from burn injuries. This was inconsistent with many studies [16-23], including studies conducted by Mehta et al. and Laham et al. [24-25]. In contrast, another study by Hegde et al. showed that *Acinetobacter* was the most common organism infecting the burn wounds [26], whereas *S. aureus* was the most common organism according to other studies by Altoparlak et al. and Erol et al. [27-28]. In our study, the resistance of *P. aeruginosa* was alarmingly high. Sensitivity to piperacillin and tazobactam in our study was 38.9% in contrast to 80.55% in the study by Saaiq et al. [16] and 11.1% in a study by Datta et al. [17]. In the study by Datta et al., *P. aeruginosa* showed the highest sensitivity to ceftazidime and meropenem with 55.6% isolates sensitive to each drug [17]. In our study, the sensitivity of *P. aeruginosa* to ceftazidime was only 8.8% and sensitivity to meropenem was 19%. After piperacillin and tazobactam, *P. aeruginosa* showed the most sensitivity to amikacin (25%) in our study. This clearly demonstrates that *P. aeruginosa* has become more resistant to drugs that were previously thought effective in the literature. Such a low sensitivity of *P. aeruginosa* demands the introduction of newer antibiotics or other methods of infection control against this organism.

*S. aureus* was the second most common organism contaminating the burn wounds in our setting; however, in another study in India by Datta et al., *S. aureus* was the most common pathogen [17]. Our finding was similar to that in another study in India by Sharma et al. where *S. aureus* was the second most common organism in the burn wounds [19]. In our study, 71.05% of *S. aureus* were methicillin-resistant. This is an approximation to the study by Saaiq et al. where 68.62% of cultures were methicillin-resistant [16], whereas 55.55% of the *S. aureus* infections were resistant to methicillin in the study by Datta et al. [17]. Gram-positive organisms may survive the initial burn injury because they may reside in the hair follicles and sweat glands and can only be eradicated by the use of surface antibiotics. These bacteria are the first to infect the burn wounds during the first 48 hours of burn injury. Therefore, measures must be taken to prevent the infection by these organisms [6, 25, 29]. MRSA isolates in our setting were completely sensitive to vancomycin and linezolid with 100% isolates sensitive to each drug. This is similar to the study by Saaiq et al. where all the S. aureus isolates were also sensitive to vancomycin and linezolid [16]. This shows that vancomycin and linezolid can be effectively given as empirical therapy for the control of MRSA infections.

The *Acinetobacter* species were the third most common microorganisms that contaminated the burn wounds in our burn ward. This is in contrast to the study by Saaiq et al. where the third most common pathogen was *S. aureus* [16] compared to coagulase-negative *Staphylococci* in the study by Datta et al. [17], and *Klebsiella* in other studies conducted in India [18-19]. Tigecycline was the most effective antibiotic against *Acinetobacter* in our study with 96% of the samples showing sensitivity to it. It showed very low sensitivity to many other the third generation cephalosporins. This is similar to the study by Hegde et al. where *Acinetobacter* showed very high resistance to all other drugs, except tigecycline for which 97.5% of the isolates were sensitive [26].

The *Klebsiella* species were the fourth most common isolate in our setting which comprised 15.19% of the isolates, whereas in the study by Saaiq et al. *Klebsiella* was the second most common bacterial isolate [16]. *Klebsiella* species in our study were most sensitive to imipenem with 44.4% isolates sensitive to it; however, in the study by Saaiq et al., piperacillin and tazobactam were found to be highly effective against *Klebsiella* with 80.95% sensitive isolates [16]. In our case, only 20.8% of the *Klebsiella* species were sensitive to piperacillin and tazobactam.

A study in India conducted by Lakshmi et al. found *E. coli* to be the second most common pathogen in burn wounds [18]. However, in our case, E. coli was not so common, accounting for only 8.23% of the total isolates. *E. coli* in our setting was the most sensitive to imipenem with 66.7% of the isolates sensitive to it, whereas amikacin and meropenem were highly effective against the E. coli in the Lakshmi et al. study [18].

The least common pathogens in our study were the *Proteus* species (4.43%), coliforms (3.8%), *Enterococcus* (0.63%), and *Enterobacter* (0.63%). In the study by Datta et al. [17], 3.33% of the cultures contained *Proteus,* whereas 17.4% had *Proteus* in the study by Lakshmi et al. [18]. The percentage of *Enterococcus* in the study conducted by Hegde et al. was 4% [26]. In a study conducted by Rajbahak et al., *Enterobacter* made up 0.93% of all the isolates, which was similar to 0.63% of *Enterobacter* isolates in our study [30].

*Pseudomonas* and *Klebsiella* was the most common bacterial combination in our study (20.59%); however, in the study by Rajbahak et al., *Pseudomonas* occurred most frequently with *Acinetobacter* (12.9%) [30].

Our study shows that resistance of all bacteria against penicillins and the third generation cephalosporins has reached a very high level.

## Conclusions

Flame burns are the most common cause of burn in females, while scald burns are more common in children. Antibiotic sensitivity pattern is everchanging. The most common bacterial isolates found in the burn wounds are *P. aeruginosa* and *S. aureus*. Piperacillin and tazobactam against *P. aeruginosa* and vancomycin and linezolid against S.aureus are highly effective and can be used as empirical therapies. The increasing antibiotic resistance of *P. aeruginosa* against the known antibiotics demands rigorous infection control, discouraging overuse and misuse of antibiotics, the invention of newer and more effective antibiotics, or adopting newer ways to control bacterial infections.
